# Immunovirological discordance among female sex workers who start antiretroviral therapy in Burkina Faso

**DOI:** 10.1186/s12879-022-07109-8

**Published:** 2022-02-03

**Authors:** Wilfried Wenceslas Bazié, Diane Yirgnur Somé, Isidore Tiandiogo Traoré, Anselme Sanon, Issouf Konaté, Souleymane Tassembedo, Ajani Ousmane Taofiki, Dramane Kania, Abdoulaye Ouédraogo, Bea Vuylsteke, Caroline Gilbert, Nicolas Meda, Abdoul Salam Ouédraogo, Nicolas Nagot

**Affiliations:** 1 Centre Muraz, Institut National de Santé Publique, 2054 Avenue Mamadou Konaté01 BP 390, Bobo-Dioulasso, Burkina Faso; 2 Institut Supérieur des Sciences de la Santé, Université Nazi Boni, Bobo-Dioulasso, Burkina Faso; 3 Department of Public Health, Institute of Tropical Medicine, Antwerp, Belgium; 4 Axe de Recherche Maladies Infectieuses et Immunitaires, Centre de Recherche du CHU de Québec-Université Laval, Québec, QC Canada; 5 Département de Santé Publique, Unité de Formation et de Recherche en Sciences de la Santé, Université Joseph Ki-Zerbo, Ouagadougou, Burkina Faso; 6 INSERM, Université des Antilles, Etablissement Français du Sang, Montpellier, France

**Keywords:** HIV-1, Antiretroviral therapy, Immunovirological discordance, Female sex workers, Burkina Faso

## Abstract

**Background:**

In people living with HIV/AIDS (PLWHA), initiation of antiretroviral therapy (ART) leads to sustained effective suppression of viral replication and increasing CD4 + T cell count. However, a fraction of ART-treated patients still fail to reach adequate CD4 + T cell number despite a suppressed viral load (VL), and this phenomenon is defined as immunovirological discordance (IVD). In Africa, several studies have reported immunovirological outcomes of antiretroviral therapy, but little is known about IVD occurrence in Female sex workers (FSW). This study aimed to assess the prevalence of IVD and associated factors among a cohort of HIV infected FSW in Burkina Faso.

**Methods:**

We conducted a cohort study from December 2003 to October 2016. Immunovirological discordance was defined as CD4 + T cell gain < 100 cells/µL despite a suppressed VL (VL < 1000 copies/mL) 12 months after ART initiation. The CD4 + T cells were counted using BD FACSCount™ System and point of care Pima™ CD4 + Analyzer. HIV-1 RNA was quantified by real-time polymerase-chain-reaction assay with the use of the ABI 7000 system. We conducted a logistic regression to identify factors associated with discordant responses.

**Results:**

Among the 123 HIV-1 infected FSW having at least 12 months follow-up on ART, 105 (85.4%) achieved HIV-1 RNA suppression. Among the latter 25 gained less than 100 CD4 + T cells within 12 months follow-up. The IVD rate was 23.8% (95%CI 16.04%–33.11%). After adjustment for age, WHO clinical stage and ART regimen including nucleoside/nucleotide reverse transcriptase inhibitors, only baseline CD4 + T cell count between 200 to 350 cells/µL (adjusted OR: 4.15; 95%CI 1.13–15.22) and 350 to 500 cells/µL (adjusted OR: 17.50; 95%CI 2.68–114.31) remain significantly associated with IVD occurrence.

**Conclusions:**

Immunovirological discordance response was common in FSW with proportions close to those observed in the general population. A diagnosis and personalized follow-up of patients who do not achieve full immune reconstitution would make it possible to avoid complications in terms of morbidity and mortality.

## Background

In people living with HIV/AIDS (PLWHA), initiation of the antiretroviral therapy (ART) lead to sustained effective suppression of viral replication, increasing CD4 + T cell count, reversal of most immunological disturbances, and reduction in risk of morbidity and mortality [1]. However, a fraction of ART-treated patients still fail to fully recover adequate CD4 + T cell number despite a suppressed viral load (VL). This phenomenon is referred to by various terms including discordant immune responders or immunovirological discordance (IVD) and has been associated with increased morbidity and mortality [2, 3]. However, there is no consensus on the time, the gain or the CD4 + T cell number to define this phenomenon. A systematic review reported that among patients with the discordant outcome, the risk of mortality ranged from 3 to 23% versus 1% to 7% for those with good immunological responses [4].

In resource constrained countries including Africa, Latin America, and Asia, the antiretroviral therapy collaboration network reported an IVD prevalence of 19.07% after six months on ART [5]. Recent studies conducted in the general population particularly in Africa reported an IVD prevalence of 14% in Burkina Faso in 2012 [6], 16% in Nigeria in 2013 [7], 29% in Rwanda in 2016 [8] and, 2.70% in Tigray, Northern Ethiopia in 2021 [9]. In current practice, IVD remains underdiagnosed or neglected for many reasons. WHO recommends that in settings where routine viral load monitoring is available, CD4 + T cell count monitoring can be stopped in individuals who are stable on ART and virally suppressed [10] and this limits the follow-up of immunological recovery. Moreover, logistical constraints with recurring stockouts of reagents and devices failure make IVD assessment difficult or often impossible to implement.

Multiple factors have been associated with IVD occurrence but none of these factors provides a full explanation for the lack of immune reconstitution [11–15]. Sociodemographic factors such as older age at ART initiation [7, 16, 17], male sex [7] have been associated with IVD. Furthermore, biological, and clinical factors such as coinfection and immune activation [18–20], CD4 + T cell count at ART initiation [5, 16, 21, 22], poor adherence [2, 23], treatment regimen (containing Zidovudine or Lamivudine/Zidovudine) [2, 24] have also been associated with IVD occurrence.

In Africa, several studies have reported immunovirological outcomes of ART but few of them have been focused on IVD assessment in Female Sex Workers (FSW). Whereas, FSW are exposed to multiple factors that could favor IVD occurrence namely, substance use, ART non-adherence, coinfections due to the high frequency of viral and bacterial sexually transmitted infections (STI) [12, 25, 26], likely increasing immune activation. The IVD rates among FSWs may highlight the need for targeted interventions to improve ART access and immunovirological monitoring to maximize the benefit of ART and limit the spread of HIV.

Knowing that the immune reconstitution that occurs during ART is essential for successful treatment of PLWHA and the lack of data on IVD occurrence among FSW, we sought to assess the prevalence of IVD and its associated factors at 12 months follow up in FSW living with HIV in Burkina Faso.

## Methods

### Study design and setting

We analyzed data from an open cohort of high-risk women followed up from December 2003 to October 2016 ('Yerelon' cohort, ANRS 1222), as described previously [27–29]. This cohort was implemented in a Clinic that provides HIV prevention and care services to professional or non-professional FSW, with strong involvement of community-based organizations (CBO) [27–29].

### Participants

Cohort of HIV infected and uninfected FSW were recruited through a network of peer educators, then enrolled after informed consent. The recruitment and inclusion have been described elsewhere [27–29]. Briefly, women working in the streets and bars of Bobo-Dioulasso were eligible for the cohort if they reported at least two transactional sex acts per week, were aged 16 years or older and were willing to undergo regular testing for HIV and STI. Other women were recruited from HIV CBO, using the same criteria. We included in this analysis all HIV infected women who fulfilled the following criteria: infected with HIV-1, aged at least 18 years, initiated antiretroviral treatment at the Yerelon clinic from December 2003 to October 2016, being on antiretroviral therapy for at least 12 months, having CD4 + T cell count results at ART initiation < 500 cells/µL, having CD4 + T cell count and viral load results at 12 months after ART initiation. The threshold of 500 cells/µL was chosen because it represents the lower limit of the normal value range of CD4 + T cells in healthy conditions [30].

### Data sources

Collected variables included socio-demographic characteristics, clinical and biological data at ART initiation, and 12 months follow-up on ART. Study participants who started ART were followed at closer intervals: weekly during the first two weeks, and monthly during the whole follow-up for clinical examination, detection of drug adverse effects, and counseling on adherence [28]. Adherence was measured by pill counts and pharmacy refills at each visit during follow up and adherence data were obtained by averaging ART adherence at each visit during the first 12 months.

### Antiretroviral therapy

ART was initiated and monitored according to national guidelines adapted from the WHO recommendation for each period [31]. The first line triple drug combination treatment included two nucleoside/nucleotide reverse transcriptase inhibitors (NRTIs) and either a non-nucleoside reverse transcriptase inhibitor (NNRTIs) or a protease inhibitor (IP) (for HIV-1 and HIV-2 coinfection). The available NRTIs molecules (Zidovudine, Lamivudine, Stavudine, Tenofovir Dixoproxil Fumarate, and Emtricitabine), NRTIs (Nevirapine and Efavirenz), and PI (Crixivan and Viracept) were used.

### Biological measurements

Blood samples were collected in EDTA-containing tubes. The CD4 + T cells were counted using BD FACSCount™ System (Becton Dickinson, USA) and point of care Pima™ CD4 + Analyzer (Alere, Germany).

BD FACSCount™ CD4 reagents are complete kits used to enumerate the absolute counts and the percentage of CD4 + T lymphocytes. Briefly, 50 µL of adequately mixed EDTA whole blood was added into the reagent tube. The tube was vortexed for 6 s and incubated for 30 min at room temperature in the workstation protected from light. Then, 50 µL of the fixative solution was added and vortexed upright for 6 s before being analyzed with the BD FACSCount instrument.

The Pima CD4 test comprises a disposable Pima CD4 test cartridge and the Pima Analyser. It is an automated, image-based immune hematology test intended for the absolute CD3 + /CD4 + T lymphocyte counts in whole blood. Succinctly, 25 µL of adequately mixed EDTA whole blood was added into the sample collector of the Pima CD4 test cartridge. Then, Insert the Pima CD4 test cartridge into the Pima Analyser to run a test.

For viral load measurement, briefly, RNA was extracted manually from plasma using the QIAamp viral RNA mini kit (QIAGEN, Courtaboeuf, France). HIV-1 RNA was quantified by real-time polymerase-chain-reaction assay with the use of the ABI 7000 system (Applied Biosystems) as described previously [32]. The lower limit of detection was 300 copies/ml (2.48 log10). During this period, the Centre Muraz laboratory participated in an external quality-control program for HIV-1 RNA quantitation organized by ANRS.

### Definitions

There is no consensus in the literature on how best to define immunologic response or immunological failure during ART and it is not clear which criteria are optimal. Aldrete et *al*. [33] exploring the longitudinal relationship between CD4 + T cell count and composite clinical event shown that CD4 + slope ≥ 100 cells/μL/year after ART initiation was associated with lower rates of AIDS, serious non-AIDS events, and death. In this analysis, in harmony with Aldrete, IVD was defined by the increase of less than 100 CD4 + T cells/μL at 12 months compared to baseline despite virological suppression (VL < 1000 copies/ml).

Good adherence to ART was defined as adherence rate > 95% and poor adherence as < 95% [34].

### Statistical analysis

Statistical analyses were performed with Epi Info™ 7. Categorical variables were expressed as frequency and/or percentage (%) whereas quantitative variables as median with interquartile range (IQR). Logistic regression by stepwise model selection was used to analyze predictors of IVD in univariate and multivariate analyses. The independent variables were included in the multivariate model if they had a p-value ≤ 0.20 on bivariate analysis. The variable age and WHO clinical stage were forced in the final model irrespective of p values. P values < 0.05 were considered statistically significant.

## Results

### Baseline characteristics of patients

**Fig. 1:**
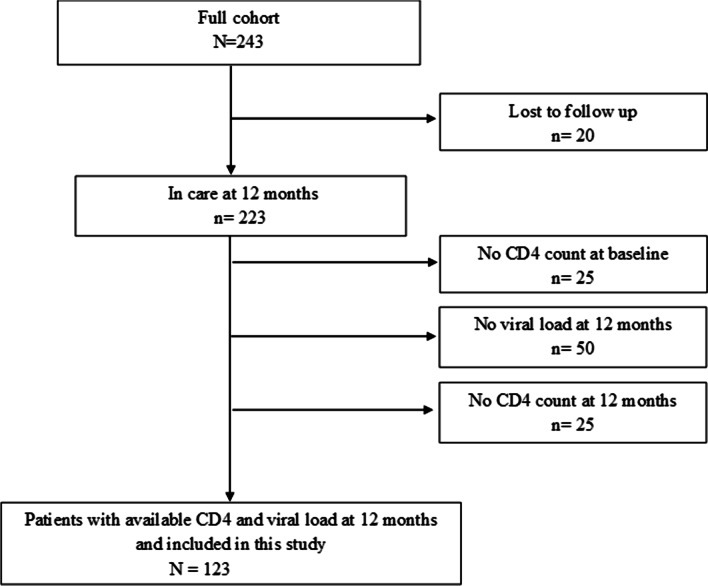
Flowchart of the Yerelon cohort patient who initiated antiretroviral therapy during study period and evaluated as part of this study

Of 243 cohort participants who started ART during the study period, 123 met inclusion criteria for this analysis (Fig. 1).

The median age of these 123 patients at ART initiation was 33 years (IQR 29–39) and 76.4% were single or widowed or divorced. At ART initiation, 52.0% were at WHO clinical stages 3 or 4, and the median CD4 + T cell count was 147 (IQR 79–200) cells/µL. Regarding ART molecules, most patients 83 (67.5%) started the Efavirenz-based regimen, and 73 (59.3%) a Zidovudine-based regimen (Table 1). Additional baseline characteristics are shown in Table 1.

**Table 1:** Yerelon cohort patient who initiated antiretroviral study participants baseline characteristics

Variable at ART initiation	All Patients (n = 123) n (%)	Patients with HIV-1 RNA suppressed at 12 months on ART (VL < 1000 copies/mL) (n = 105) n (%)	Immunologic responder at 12 months on ART (CD4 + T cell recovery ≥ 100 cells) (n = 86) n (%)	Patients with immunovirological discordance at 12 months (n = 25) n (%)
Age: median (IQR) years	33.00 (29.00–39.17)	33.02 (30.01–39.02)	32.93 (28.5–38.80)	33.00 (31.00–39.23)
< 30	31 (25.20)	25 (23.81)	23 (26.74)	4 (16.00)
30–39	65 (52.85)	58 (55.24)	46 (53.49)	15 (60.00)
≥ 40	27 (21.95)	22 (20.95)	17 (19.77)	6 (24.00)
Education level				
None	52 (42.28)	45 (42.86)	38 (44.19)	9 (36.00)
Primary school	36 (29.27)	31 (29.52)	23 (26.74)	11 (44.)
Secondary school	35 (28.45	29 (27.62)	25 (29.07)	5 (20.00)
Marital status (couple life)				
Yes	29 (23.58)	23 (21.90)	21 (24.42)	4 (16.00)
No	94 (76.42)	82 (78.10)	65 (75.58)	21 (8400)
WHO clinical stage				
1	17 (13.82)	13 (12.38)	12 (13.95)	3 (12.00)
2	42 (34.15)	35 (33.33)	29 (33.72)	8 (32.00)
3 and 4	64 (52.03)	57 (54.29)	45 (52.33)	14 (56.00)
Body mass index: median (IQR)	20.60 (18.55–22.72)	20.58 (18.13–22.55)	20.38 (18.24–22.37)	20.76 (18.55–23.51)
< 18,5	29 (23.58)	28 (26.67)	23 (26.74)	6 (24.00)
18,5–25	83 (67.48)	67 (63.81)	57 (66.28)	15 (60.00)
> 25	11 (8.94)	10 (9.52)	6 (6.98)	4 (16.00)
CD4 + T cell count at ART initiation: Median (IQR)	147 (79–200)	139 (74–200)	136 (75–192)	238 (100–384)
< 200	90 (73.17)	77 (73.33)	70 (81.40)	11 (44.00)
200–349	23 (18.70)	19 (18.10)	14 (16.28)	7 (28.00)
350 -500	10 (8.13)	9 (8.57)	2 (2.33)	7 (28.00)
HIV-1 Viral load (log 10copies/ml): median (IQR) (n = 52)	5.20 (4.82–5.52)	5.26 (4.85–5.58)	5.18 (4.82–5.54)	5.43 (5.28–5.65)
< 3 log10	2 (3.85)	2 (4.17)	2 (4.76)	-
3–5 log10	16 (30.77)	13 (27.08)	13 (30.95)	1 (12.50)
> 5log10	34 (65.38)	33 (68.75)	27 (64.29)	7 (87.50)
ART NNRTIs initiated				
Nevirapine	38 (30.89)	30 (28.57)	28 (32.56)	5 (20.00)
Efavirenz	83 (67.48)	73 (69.52)	56 (65.12)	20 (80.00)
Protease inibitor	2 (1.63)	2 (1.90)	2 (2.33)	
ART NRTIs initiated				
Tenofovir	18 (14.63)	15 (14.29)	11 (12.79)	6 (24.00)
Zidovudine	73 (59.35)	64 (60.95)	49 (56.98)	17 (68.00)
Stavudine	32 (26.02)	26 (24.76)	26 (30.23)	2 (8.00)
ART Adherence: median (IQR)	97.00 (92.5–99.40	97.00 (93.5–99.65)	97.00 (92.00–99.40)	96.40 (95.30–100)
< 95%	45 (36.59)	37 (35.24)	35 (40.70)	6 (24)
≥ 95%	78 (63.41)	68 (64.76)	51 (59.30)	19 (76)

*ART* antiretroviral therapy, *IQR* interquartile range, *NNRTIs* non-nucleoside reverse transcriptase inhibitors, *NRTIs* nucleoside/nucleotide reverse transcriptase inhibitors

### *FSW virologic response to treatment and CD4* + *T cells recovery*

At 12 months follow-up of 123 ART-treated participants, 105 (85.4%) had attained VL suppression (VL < 1000 copies/mL) (Table 2). Compared to those with unsuppressed VL, no significant difference was observed with patients who have suppressed VL at 12 months respectively in terms of baseline median age (30.8 (IQR 27.69–40.15) vs 33.02 (IQR 30.01–39.02) p = 0.5694), baseline median CD4 + T cell count (158 (IQR 130–214) vs 139 (IQR 74–200) p = 0.2144), and baseline median VL (4.80 (IQR 4.02–4.85) vs 5.26 (IQR 4.85–5.58) p = 0.0687) (Table 2).

**Table 2:** Virological and immunological treatment responses at 12 months among Yerelon cohort patients who initiated antiretroviral

	Viral Load		Total	P-value
	< 1000 copies /mL n (%)	≥ 1000 copies /mL n (%)		
CD4 + T cell increase				
< 100 cells	25 (23.81)	12 (66.67)	37	
≥ 100 cells	80 (76.19)	6 (33.33)	86	0.0003
Total	105	18	123	
Baseline age: median (IQR)	33.02 (30.01–39.02)	30.8 (27.69–40.15)		0.5694
Baseline CD4 + T cell count: median (IQR)	139 (74–200)	158 (130–214		0.2144
HIV-1 Viral load (log 10copies/mL): median (IQR)	5.26 (4.85–5.58	4.80 (4.02–4.85		0.0687

Concerning immunologic response based on CD4 + T cell count recovery, four patients lost CD4 + T cells, and the median increase of CD4 + T cells among 119 patients who gained was 167 cells/µL (IQR 89–286). Irrespective of VL suppression, 86 patients (69.9%) achieved a CD4 + T cells gain greater than 100 cells after 12 months of treatment (Table 2) with a median gain of 218 CD4 + T cells (IQR 160–311). These patients seem to have significantly fewer baseline CD4 + T cells compared to others (136 (IQR 75–192) vs 181 (IQR 109–343), p = 0.0046).

When we assessed immunologic recovery as a function of VL suppression status (VL < 1000 copies/mL), the IVD rate was 23.8% (95%CI 16.04%–33.11%).

At 12 months follow-up, 78 (63.4%) patients achieved good adherence with a median ART adherence rate of 98.6% (IQR 97.0–100). The adherence rate was 97.0% (IQR 93.5–99.6) for those with suppressed VL and 96.4% (IQR 95.3–100) for those who presented an IVD. Table 1 presents the baseline characteristics of patients who obtained a plasma HIV RNA level < 1000 copies/mL at 12 months and who presented an IVD.

### Factors associated with immunovirological discordance

We next evaluated factors associated with IVD in 105 patients who obtained VL suppression. In bivariate analysis, patients with baseline CD4 + T cell count between 200 and 350 cells (OR: 3.50; 95%CI 1.13–10.83) and between 350 and 500 cells (OR: 21.00; 95%CI 3.85–114.51) were more likely to present IVD at 12 months on ART compared to those with CD4 + T cell count below 200 cells/µL (Table 3). Besides, participants who had ART regimens that included stavudine (OR: 0.12; 95%CI 0.02–0.74) were less at risk to present IVD compared to those initiated with ART regiments that included tenofovir (Table 3).

**Table 3:** Factors associated with immunovirological discordance at 12 months on antiretroviral therapy at univariate and multivariate logistic regression

Variable	n (%) of immunovirological discordance	Odds Ratio (95% CI)	*P*	Adjusted Odds Ratio (95% CI)	*P*
Age (years)			0.3540		0.5032
< 30	4 (16.00)	1		1	
30–39	15 (60.00)	1.83 (0,54–6.20)		1.55 (0.39–6.10)	0.5323
≥ 40	6 (24.00)	1.97 (0.47–8.16)		1.76 (0.33–9.28)	0.5050
Marital status (couple life)			0.4166		
No	4 (16.00)	1			
Yes	21 (8400)	0.61 (0.19–2.00)			
BMI Class			0.3658		
< 18,5	6 (24.00)	1			
18,5–25	15 (60.00)	1.06 (0.36–3.08)			
> 25	4 (16.00)	2.44 (0.52–11.56)			
WHO clinical stage			0.8640		0.5306
1	3 (12.00)	1		1	
2	8 (32.00)	0.99 (0.22–4.48)		0.82 (0.13–4.98)	0.8300
3 and 4	14 (56.00)	1.08 (0.26–4.51)		1.28 (0.24–6.65)	0.7716
CD4 + T cell count (cells/µl)			0.0001		0.0012
< 200	11 (44.00)	1		1	
200–349	7 (28.00)	3.50 (1.13–10.83)		4.15 (1.13–15.22)	0.0317
350–500	7 (28.00)	21.00 (3.85–114.51)		17.50 (2.68–114.31)	0.0028
ART NNRTIs initiated			0.4313		
Nevirapine	5 (20.00)	1			
Efavirenz	20 (80.00)	1.89 (0.63–5.61)			
Protease inhibitor		–			
ART NRTIs initiated			0.0170		0.1800
Tenofovir	6 (24.00)	1		1	
Zidovudine	17 (68.00)	0.54 (0.16–1.75)		1.19 (0.27–5.29)	0.8177
Stavudine	2 (8.00)	0.12 (0.02–0.74)		0.28 (0.04–2.11)	0.2183
ART Adherence			0.2495		
< 95%	6 (24)	1			
≥ 95%	19 (76)	2.00 (0.72–5.56)			

On the other hand, patients who initiated antiretroviral regimens that included efavirenz as NNRTI tended to be more likely to have IVD after 12 months (OR: 1.89; 95%CI 0.63–5.61). Other factors like age, body mass index, and WHO clinical stage were not associated with IVD occurrence at 12 months on ART (Table 3).

At multivariate analysis, after adjustment for age, WHO clinical stage, and ART regimen including NRTIs, only baseline CD4 + T cell count between 200 and 350 cells/µl (adjusted Odds Ratio (aOR): 4.15; 95%CI 1.13–15.22) and between 350 and 500 cells/µl (aOR: 17.50; 95%CI 2.68–114.31) remain significantly associated with IVD occurrence (Table 3).

## Discussion

The frequency of IVD in our population of FSW was 23.81% after 12 months of follow-up on ART. This finding was similar to previous reports in the general population which noticed that 10–30% of all HIV-infected patients do not achieve optimal immune reconstitution despite suppression of viral replication [2, 5, 8, 15, 16, 35]. It is important to note that there is no consensus in the literature on how best to define IVD. Some studies define IVD as a failure to achieve a pre-specified absolute CD4 + T cell count (threshold) at a particular time point. Most of these studies focus on a threshold (i.e. < 200 or < 350 cells/μL) derived from prior guidelines that determined the timing of ART initiation and risk of opportunistic infections [9, 14, 30]. Others require a pre-specified increase in CD4 + T cell count irrespective of the initial value in percentage or a threshold value to define IVD [8, 14, 33]. Likewise, the suppressed or undetectable viral load threshold changes according to the studies. This multitude of definitions makes it difficult to compare the different studies in addition to the fact that they are carried out in different contexts and populations. There is a need for agreement on defining IVD for standardization across the different ways of identifying it.

In this study, a discordant response was associated with a higher CD4 + T cell count above 200 cells/µl at ART initiation. Other studies have reported similar findings to ours, with higher pre-therapy CD4 + T cell count being associated with smaller gains in CD4 + T cell count at 12 months [5, 16, 23]. Tuboi [5] and Moore [2] reported that increases in CD4 + T cell count after initiation of therapy might be greater in individuals with lower CD4 + T cell count at therapy initiation.

Contrariwise, some studies reported that a low baseline CD4 + T cell count was predictive of immunological discordant treatment responses [9, 36, 37] while some others found no association between baseline CD4 + T cell count and IVD [8]. After ART initiation, the initial increase in CD4 + T cell count is usually observed in the first 3–6 months [38]. Several factors including impaired bone marrow hematopoietic function and decreased proliferative capacity, lower thymic output, dysfunction in some cytokines expressions, and CD4 + T cells destructions may have an important role in the achievement of optimal immune reconstitution [13–15].

Another contributing factor that could particularly expose FSW to IVD and not assessed in our study is coinfection with other pathogens which is prevalent in Africa [39, 40]. Numerous studies have found that hepatitis B virus, hepatitis C virus, and cytomegalovirus coinfections were associated with poor CD4 + T cells immune recovery in HIV‐1‐infected individuals on ART [13, 41, 42]. The mechanism by which coinfections may have deleterious effects on CD4 + T cell count recovery is linked to its contribution to immune activation. Hunt et al. [19] found that with every 5% increase in the percentage of activated CD8 T cells, the CD4 + T cell count decreased by 35 cells/µL after 3 months of ART. Over study indicated that T cell activation driven by monocyte activation demonstrated by Soluble CD14 and soluble CD163 levels is associated with poor immune recovery in HIV‐1 infected individuals [43].

In contrast to most of the studies, age was not associated with IVD in our study. In the evaluation of immune recovery, younger age was described to predict greater early CD4 + T cell gain and supporting the importance of thymic function [13, 17, 44, 45]. Our participants were relatively younger with a median age of 33 years. In fact, older patients were more likely to experience poorer immunologic responses that could be potentiated by HIV induced immunosenescence [13, 23]. Moreover, the normal aging process leads to the gradual change of thymic tissue into fatty tissue and this may also explain why older people are at higher risk of incomplete immune restoration [14].

Unexpectedly, patients that initiated therapy with a tenofovir containing regimen were more likely to develop IVD than patients on a Zidovudine or stavudine containing regimen. This could probably be explained by the fact that tenofovir was introduced when the threshold for CD4 count to initiate ARV treatment was 350 cells. In addition, the relatively small number of patients on tenofovir compared to other molecules could also explain these results. However, evidence on nucleoside/nucleotide reverse transcriptase inhibitors effect on CD4 + T cells recovery was controversial [35, 46, 47]. Most of our patients were on a regimen including a NNRTI rather than a protease inhibitor. However, compared to NNRT, protease inhibitors based regimens were shown to modulate the activation of peripheral blood CD4 + T cells, decrease their susceptibility to apoptosis therefore promote their increase [48].

Contrary to Abrogoua et al. [49] who noticed that adherence to ART is an important predictor of immunological response, no significant association between IVD and ART adherence was observed. We think that in the lack of an objective method for adherence assessment (drug assay in the blood), it is difficult to attribute IVD to an adherence problem.

To date, no antiretroviral or immune system-based strategy has yet demonstrated a clinically meaningful outcome in the treatment of IVD. Some data support less frequent CD4 + T cell monitoring in clinically stable virally suppressed patients and suggest that routine CD4 + T cell monitoring for this population may be unnecessary [50, 51]. For us, rather than suppressing the CD4 + T count in all patients with suppressed viral load, we believe that for personalized medicine it would be desirable to maintain annual monitoring of the CD4 + T cell count in patients with IVD. Its measure would not only permit to discontinue but also to restart the prophylaxis for opportunistic infections.

Our study has some limitations. Some findings could be explained by the relatively small size of the sample, which does not ensure sufficient power to be able to extrapolate the statistical analysis results to the overall population. We did not have baseline HIV-1 RNA measurements for more than half of the patients. These results would have made it possible to better assess the evolution of viral load under ART. The CD4 + T cell count was not performed with the same counting device for all patients. A study showed a 2% difference between the results of the CD4 + T cell count performed with the BD FACSCount device and the Pima analyzer from a threshold of 350 cells/µL [52]. As a result, patients may be wrongly considered to be in discordance.

## Conclusion

Despite the difficult context linked to the lack of a consensual definition of IVD and the period over which it must be evaluated, our results show a frequency of IVD in FSW comparable to those observed in the general population. Despite several pejorative considerations, the monitoring set up in the Yerelon cohort makes it possible to obtain the same rates of IVD in FSW as in the general population. A better understanding of IVD occurrence and associated factors may lead to better therapies to improve the level of immune reconstitution and ultimately the possible development of adjuvants that reverse the immunologic perturbations caused by HIV infection and that persist despite ART induced viral suppression.

## Data Availability

The datasets used and/or analysed during the current study are available from the corresponding author on reasonable request.

## Abbreviations

ART: Antiretroviral therapy
VL: Viral load
IVD: Immunovirological discordance
FSW: Female sex workers
STI: Sexually transmitted infections
CBO: Community-based organizations
NRTIs: Nucleoside/nucleotide reverse transcriptase inhibitors
NNRTIs: Non-nucleoside reverse transcriptase inhibitors
IP: Protease inhibitor
IQR: Interquartile range
OR: Odds ratio
aOR: Adjusted OR
